# Neonatal resuscitation skills acquisition among healthcare providers after Helping Babies Breathe simulation training using improved tools across two regions in Tanzania

**DOI:** 10.1186/s41077-025-00338-2

**Published:** 2025-03-01

**Authors:** Florence Salvatory Kalabamu, Vickfarajaeli Daudi, Robert Moshiro, Benjamin Kamala, Paschal Mdoe, Dunstan Bishanga, Hege Ersdal, Rose Mpembeni

**Affiliations:** 1 Muhimbili University of Health and Allied Sciences, Dar Es Salaam, Tanzania; 2 Hubert Kairuki Memorial University, Dar Es Salaam, Tanzania; 3 Muhimbili National Hospital, Dar Es Salaam, Tanzania; 4 Haydom Lutheran Hospital, Manyara, Tanzania; 5 Ifakara Health Institute, Dar Es Salaam, Tanzania; 6 Stavanger University Hospital, Stavanger, Norway; 7 University of Stavanger, Stavanger, Norway

**Keywords:** Helping Babies Breathe, Neobeat, Neonatalie live, Safer births, Simulation

## Abstract

**Introduction:**

Neonatal mortality is high in middle- and low-income countries, including Tanzania. Most of these deaths are preventable and linked to suboptimal quality of care. In this study, we assessed neonatal resuscitation skills acquisition after a 1-day Helping Babies Breathe (HBB) simulation training using improved tools and associated factors among healthcare providers in 12 facilities in Tanzania.

**Methods:**

A cross-sectional study was conducted among healthcare providers working in the labor wards in selected health facilities. The training was conducted in situ using the HBB second edition curriculum with improved simulation tools (Neonatalie Live simulator, NeoBeat heart rate meter, and Upright resuscitator). After training, skills acquisition was evaluated using Objectively Structured Clinical Evaluation. Participants who scored an average of 75% or above were considered passing. Descriptive statistics were used to determine the proportion of staff who passed the evaluation by different demographic categories. One-way analysis of variance was used to compare mean scores among demographic categories. Factors associated with neonatal resuscitation skills acquisition were analyzed using modified Poisson regression.

**Results:**

A total of 481 participants were enrolled in the study. Among these, 420 (87.3%) passed the skills evaluation on the first attempt. The overall mean skills score was 92.4%. In bivariable analysis, health facility level, region, age, and experience working in the labor ward were associated with passing skills evaluation on the first attempt. However, after controlling other variables in a multivariable model, none of the factors showed a statistically significant association.

**Conclusion:**

In**-**situ, HBB simulation training using improved training tools effectively imparts neonatal resuscitation skills among healthcare providers. Participants learned skills similarly regardless of their different demographic characteristics, including level of education and working experience. Due to its potential to impart skills, frequent simulation training using improved tools may be considered for scaling up in other health facilities.

## Introduction

Globally, there has been a significant decrease in under-5 and infant mortality rates due to coordinated efforts and individual country commitments [1–3]. A similar achievement has not been realized with neonatal mortality, whereby almost 47% of all under-5 deaths occur in the neonatal period [4]. In 2020, the World Health Organization (WHO) estimated that around 2.3 million neonates die globally, with almost 95% occurring in low- and middle-income countries (LMIC) [5].

In Tanzania, under-5 mortality has been reduced by around 70% from 162 per 1000 live births in 1999 to 43 per 1000 live births in 2022, while neonatal mortality has been reduced by 40% only during the same period [6]. The current Tanzania Demographic and Health Indicators Survey has estimated a neonatal mortality rate of around 24 deaths per 1000 live births, which is higher than the overall global estimate of 18 deaths per 1000 live births [5, 6]. Tanzania is not on track to meet the Sustainable Development Goal (SDG) number 3.2 aiming to reduce newborn and child mortality, with focusing on reducing the neonatal mortality rate to not more than 12 deaths per 1000 live births before 2030 [2].

In Tanzania, like other parts of the world, the main three culprits of neonatal mortality are birth asphyxia, sepsis, and complications of prematurity [7–9]. Almost 50% of these deaths occur in the 24 h after birth, and up to 75% in the first week of life, linked to suboptimal quality of care during the intrapartum period and immediately after delivery. Notably, interventions aimed at improving intrapartum and immediate care of the neonate after birth have significantly reduced neonatal mortality [10–12].

Improving and sustaining neonatal care skills among healthcare providers is essential to ensuring survival [10, 13, 14]. These skills include providing immediate basic neonatal care and identifying neonates who need more support, including lifesaving interventions such as bag-mask ventilation within the “golden minute” [15–17]. Research findings from Helping Babies Breathe (HBB) training and practice in Tanzania showed that for every 30-s delay in starting to ventilate a non-breathing neonate, the risk of death/morbidity increases by 16% [15]. This highlights the urgency to provide competent care after birth and the continuous importance of skillful healthcare workers [16–18]. This can be effectively done with more focus on strengthening essential competencies for midwives in newborn care immediately after birth including essential newborn care practices, detection of complications, and stabilization of emergencies [16]. However, an HBB training evaluation conducted in Tanzania demonstrates a skills gap in neonatal resuscitation and skills decay over time, jeopardizing the safety and care of neonates [17].

In response to this gap, the Safer Births Bundle of Care (SBBC) is being implemented in five regions in Tanzania [18] aiming to improve perinatal outcomes by utilizing innovative training tools in combination with the HBB second edition [17]. Moreover, the bundle utilizes training and clinical data to guide continuous quality improvement activities and identify skills gaps that need to be addressed. This study aims to describe neonatal resuscitation skills acquisition and associated factors among healthcare providers following the initial SBBC training in selected facilities in the Geita and Shinyanga regions.

## Methods

### Study sites, design, and population

This cross-sectional study was conducted in 12 health facilities in Shinyanga and Geita regions in the northern-western part of Tanzania (6 facilities from each region). This study design was considered more appropriate to assess neonatal resuscitation skills acquisition immediately after simulation training. Government-owned facilities were purposely selected based on a high burden of maternal and perinatal mortality, high volume of delivery per year, and the absence of other similar interventions during the study period. Both could provide Comprehensive Emergency Obstetric and Newborn Care (CEmONC) packages that include management of bleeding after birth, infections, prolonged or obstructed labor, eclampsia, and birth asphyxia.

In the Tanzanian health system context, healthcare service delivery follows a defined hierarchy; dispensaries are the lowest level of facilities close to the community that are responsible for providing primary healthcare services on an outpatient basis. In that order, subsequent facility levels are Health Centers and district, Regional, Zonal, and National referral hospitals. Patients are managed or referred to the next-level facilities following this hierarchy, depending on the disease severity, expertise, and availability of services.

The study population comprised healthcare providers working in the labor ward and obstetric theaters during the initial SBBC training. These included nurses/midwives, and doctors. In the Tanzanian context, nurses are divided into three cadres according to number of years of preservice training: “Enrolled” nurses are the lowest cadre with 2 years of formal training and are awarded a certificate, while “registered” nurses include nurses with three or more years of formal training with bachelor’s degree or a diploma in nursing. In Tanzania’s nursing curriculum, all nurses and midwives are trained on essential competencies for midwives according to the International Confederation of Midwives [16]. Certified midwives are trained further in advanced midwifery practices. This was done to facilitate task sharing as a result of inadequate certified midwives. For this research, doctors are registered medical practitioners regardless of their level of training. These include medical specialists with postgraduate degrees in specific clinical areas. These medical officers are graduate general practitioners, assistant medical officers with advanced diplomas in clinical practice, and clinical officers who have ordinary diplomas in clinical medicine. Each level has a specific job description based on the level of training and experience.

### Improved neonatal resuscitation training tools

The improved, innovative tool used for neonatal resuscitation training was NeoNatalie Live. This smart simulator provides the learner with objective feedback on the most important aspects of her/his performance during bag-mask ventilation skills practice [19]. The simulator can be connected to a tablet or a phone by Bluetooth. The information on the tablet guides the learner on which aspects of the neonatal resuscitation process need improvement in subsequent training, such as ventilation rate, pressure, face-mask seal, and positioning of the head to open the airway. Another innovative tool is the Upright resuscitator, designed to seal the mouth and handle the nose during resuscitation easily. Finally, the NeoBeat (neonatal heart rate meter) provides a continuous display of neonatal heart rate to guide resuscitation [19]. These tools were innovated in the Safer Births collaborative project (www.saferbirths.com) and distributed by Laerdal Global Health [20].

### SBBC Training cascade

#### Training of national facilitators

Fifteen members from the professional associations, the Paediatrics Association of Tanzania (PAT) and Tanzania Midwives Association (TAMA) were selected as national facilitators in collaboration with the respective Regional Health Management Teams (RHMT) and the study team. The selection criteria were based on their experience in the labor ward and obstetric theatre, as well as their track records in facilitating health-related training and supportive supervision in their respective regions.

Training of the national facilitators was undertaken for 2 weeks by SBBC team members and SAFER experts in simulation (Stavanger, Norway). The facilitators were trained in SimBegin and EU Sim Level 1 courses [21]. This was to enable them to be national facilitators for SBBC simulation training, including facilitating facility-based simulation training, the use and maintenance of SBBC tools, and skills assessment using Objectively Structured Clinical Evaluation (OSCE) tools.

#### Training of facility champions

Two facility champions from each facility were selected by the SBBC team in collaboration with the RHMT, District Health Management Teams (DHMT), and facility administration based on their ability to motivate their colleagues to participate in trainings using SBBC tools. The national facilitators trained facility champions for 6 days on facilitating in-situ simulations, using SBBC clinical and training tools, mentorship, and facility-based data for continuous quality improvement. Facility champions were expected to motivate their colleagues to participate in regular low-dose, high-frequency simulation training for in-facility training. Additionally, they were prepared to support the national facilitators on baseline facility-based training and continuous mentorship.

#### Initial facility-based training

A 1-day neonatal resuscitation simulation training was conducted in the selected facilities in November 2021 (Geita) and January 2022 (Shinyanga). National facilitators facilitated this training with the respective facility champions using the HBB curriculum designed by the American Academy of Paediatrics (AAP) 2nd Edition [22], utilizing the innovative Safer Births tools.

Participants were trained on how to provide routine care for a normal neonate and how to resuscitate a neonate who is not initiating spontaneous breathing using innovative tools. They were provided with time to practice using the tools individually and in groups and role plays. To ensure familiarization with the tools and simulation debriefing, they were given time to ask questions on the functionalities of the tools, how to troubleshoot them in case of malfunctioning, and how to conduct simulation. Additionally, they evaluated their resuscitation skills using feedback from the NeoNatalie Live displays and their peers in role plays.

### Skills evaluation and data collection procedures

After participants had practiced and developed confidence in using the training tools, their skills in neonatal resuscitation were assessed using the HBB-designed OSCE tools 2nd edition. The evaluation was planned in two stations: one for OSCE-A evaluation, which evaluates skills in preparation for delivery and routine immediate neonatal care, and OSCE-B, which involves evaluation of complex skills in the resuscitation of a neonate who fails to establish spontaneous breathing by following recommended sequence from physical stimulation, suction when indicated, and effective ventilation using bag and mask [23].

The national facilitators evaluated participant’s skills using the electronic version of the OSCEs in the Open Data Kit (ODK). After completing the scoring, data were saved automatically in the central database at Haydom Lutheran Hospital. Those who did not pass skills evaluations on the first attempt were given other chances to practice and repeat skills evaluations until they had passed them. This was to ensure that every participant achieved the minimum required neonatal resuscitation skill at baseline.

### Data analysis

Data were analyzed using Statistical Package for Social Sciences (SPSS) version 23 (IBM; Armonk, NY, USA) software. Baseline characteristics in each category were summarized as frequencies and respective percentages. OSCE scores were checked for normal distribution and there was no significant departure from normality. Therefore, mean scores for OSCE-A and B were calculated with respective standard deviations. Additionally, since both OSCEs have equal weights, the overall skills performance for every participant was obtained by calculating the average of OSCE-A and B.

Participants who scored an average of 75% or above on the first attempt were considered to have passed the evaluation per the American Academy of Paediatrics guideline on implementing HBB [3]. Therefore, the proportion of those who passed was determined using a frequency distribution and respective percentages. A chi-square test was performed to assess the differences in the frequency distribution of the passing status in each demographic characteristic variable. Furthermore, skills performance within and across different healthcare providers’ characteristics were compared using an independent sample *t*-test for variables with two groups. In comparison, one-way analysis of variance (ANOVA) was used for variables with more than two groups. Post hoc analysis and pairwise comparison were performed using the Bonferroni test for variables that showed significant differences in ANOVA.

### Factors associated with passing neonatal resuscitation skills evaluation on the first attempt

The factors associated with passing skills evaluation on the first attempt were determined using modified Poisson regression analysis. This model was used instead of binary logistic regression because, in this study, the probability of passing skills evaluation was higher than 15% [24]. Therefore, the estimates used in this case are the prevalence ratio instead of the odd ratios [25]. Variables with a *p*-value of ≤ 0.2 in bivariate analysis were included in the multivariate model. Adjusted prevalence ratios and respective 95% confidence intervals were calculated for each independent variable. Factors with *p*-values less or equal to 0.05 in the multivariate model were considered statistically significant.

## Results

A total of 481 healthcare providers were enrolled in the study, 421 (87.5%) of whom were nurses. Most participants were lower and middle cadres, while those with university degrees (graduates) comprised only 9.8% (Table 1).

**Table 1:** Basic demographic characteristics of study participants (*N* = 481)

Variable	Frequency	Percentage
Profession of the healthcare provider		
Nurses	421	87.5
Doctors	60	12.5
Sex		
Male	185	38.5
Female	296	61.5
Level of education		
Certificate	196	40.7
Diploma	238	49.5
Graduates (degree)	47	9.8
Age (years)		
20–30	135	28.1
31–40	243	50.5
41–50	63	13.1
51–60	40	8.3
Working experience (years)		
Up to 5 years	146	30.4
6–10	221	45.9
11–15	53	11.0
16–20	22	4.6
More than 20	39	8.1
Experience working in labour ward		
Up to 5	358	74.4
6–10	99	20.6
More than 10	24	5.0
History of previous training in HBB		
No	2	0.4
Yes	479	99.6

### Neonatal resuscitation skills acquisition

The mean skills scores were 92.2% and 92.7% for OSCE A and B, respectively, with an overall average skill score of 92.4%. Table 2 shows the average skills scores for both OSCEs in the 12 facilities. Four hundred twenty (87.3%) participants passed the skills evaluation on the first attempt. The rest passed the evaluation on the second attempt after receiving feedback from the evaluators. A comparison of demographic characteristics for those who passed on the first versus those who did not pass is presented in Table 3. Health facility level, region, age, and experience working in the labor ward were individually associated with passing skills evaluation on the first attempt.

**Table 2:** Neonatal resuscitation skills performance in health facilities (*N* = 481)

Region	Name of the healthcare facility	Number of participants	Average scores	Std. deviation
Geita	Chato District Hospital	42	96.01	3.507
	Geita Regional Referral Hospital	79	96.37	3.183
	Katoro Health Center	29	92.14	4.957
	Masumbwe District Hospital	25	93.16	4.446
	Uyovu Health Center	37	85.59	5.362
	Nzera District Hospital	21	88.12	5.971
Shinyanga	Bugarama District Hospital	13	89.62	3.471
	Bulungwa Health Center	21	87.43	7.102
	Kahama District Hospital	101	93.47	4.534
	Shinyanga Regional Referral Hospital	60	88.78	6.270
	Kambarage Health Center	37	92.36	4.725
	Nindo Health Center	16	100.00	.000
	Total	481	92.41	5.932

**Table 3:** Skills performance level by demographic characteristics of participants (*N* = 481)

Participants characteristics	Passed on first attempt? *n* (%)		*p*-value*
Region	No	Yes	
Geita	22 (9.4)	211(90.6)	0.04
Shinyanga	39 (15.7)	209 (84.3)	
Facility level			
Health Center	8 (6)	126 (94)	0.01
District hospital	35 (16.8)	173 (83.2)	
Regional referral Hospital	18 (12.9)	121 (87.1)	
Work experience (years)			
up to 5 years	16 (11)	130 (89)	0.01
6–10	25 (11.3)	196 (88.7)	
11–15	6 (11.3)	47 (88.7)	
16–20	2 (9.1)	20 (90.9)	
More than 20	12 (30.8)	27 (69.2)	
Experience working in the labor ward			
Up to 5	45 (12.6)	313 (87.4)	0.03
6–10	9 (9.1)	90 (90.9)	
More than 10	7 (29.2)	17 (70.8)	
Age (years)			
20–30	17 (12.6)	118 (87.4)	0.1
31–40	25 (10.3)	218 (89.7)	
Above 40	19 (18.4)	84 (81.6)	
Profession of the healthcare provider			
Nurses	57 (13.5)	364 (86.5)	0.1
Clinicians	4 (6.7)	56 (93.3)	
Level of education			
Certificate	34 (17.3)	162 (82.7)	0.04
Diploma	23 (9.7)	215 (90.3)	
Graduates	4 (8.5)	43 (91.5)	
Sex			
Male	18 (9.7)	167 (90.3)	0.1
Female	43 (14.5)	253 (85.5)	

*Chi square test

### Comparison of average skills scores within different demographic characteristics

In assessing the average skills scores for the different demographic characteristics, there was a significant difference in scores across education level, working experience, and health facility level (Table 4).

**Table 4:** Skills performance among healthcare providers by demographic characteristics (*N* = 481)

Variable	Number of participants	Mean score	Std. deviation	*p*-value
Regions				
Geita	234	92.9	5.9	0.06^Ϯ^
Shinyanga	247	91.9	5.9	
Sex				
Male	185	92.79	5.832	0.3^Ϯ^
Female	296	92.18	5.991	
Age				
20–30	135	92.79	6.085	0.1*
31–40	243	92.68	5.669	
Above 40	103	91.29	6.251	
Profession of the HCW				
Nurses	421	92.39	5.874	0.87^Ϯ^
Clinicians	60	92.53	6.377	
Level of education				
Certificate	196	91.45	6.157	0.01*
Diploma	238	93.16	5.538	
Graduates	47	92.64	6.453	
Working experience				
up to 5 years	146	93.34	5.622	0.04*
6–10	221	92.31	5.768	
More than 10	114	91.43	6.485	
Experience working in the labor ward (years)				
Up to 5	358	92.48	5.910	0.22*
6–10	99	92.66	5.677	
More than 10	24	90.40	7.097	
Health facility level				
Health center	134	90.66	6.712	< 0.001*
District hospital	208	93.08	5.037	
Regional referral hospital	139	93.10	6.064	

*One-way ANOVA

^Ϯ^Independent sample *t*-test

### Comparison of average skills performance of healthcare providers in relation to the level of education, working experience, and facility level

On pairwise comparison of average scores within demographic groups with significant differences in scores, middle-level education HCW (with diploma) scored significantly higher than lower-level education HCW (certificate) (*p* = 0.009). There was no statistical difference in other group pairs within the level of education categories. Generally, mean scores decreased with increasing working experience. HCWs with up to 5 years of working experience scored higher than those with more than 10 years of working experience (*p* = 0.03) (Table 5). The difference was not statistically significant in other pairs within the working experience categories. HWCs in higher facility levels had higher mean skills scores compared to lower levels. Notably, HCWs working in district hospitals and regional referral hospitals had significantly higher scores compared to those working in health centers; *p* = 0.001 and 0.002, respectively. However, there were no significant differences in mean scores between district hospitals and regional referral hospitals (Table 5).

**Table 5:** Pairwise comparison of skills performance across different variable groups

**Variable groups**		**Mean Difference **	**Std. Error**	**P value**
**Level of education**				
Certificate	Diploma	-1.701^*^	.568	0.009
	Graduates	-1.184	.956	0.649
Diploma	Graduates	.517	.940	1.000
**Working experience (years)**				
≤ 5 years	6-10	1.030	0.630	0.307
	More than 10	1.901^*^	0.738	0.031
6-10	More than 10	.871	0.681	0.604
**Facility level**				
Health center	District Hospital	-2.427^*^	0.647	0.001
	Regional referral Hospital	-2.440^*^	0.707	0.002
District Hospital	Regional referral Hospital	-0.013	0.640	1.000

^*^Statistically significant

### Factors associated with passing neonatal resuscitation skills evaluation on the first attempt

In bivariable analysis, health facility level, region, age, and experience working in the labor ward were associated with passing the skills evaluation on the first attempt. However, after controlling for other confounders, these factors were not independently associated with passing the skills evaluation on the first attempt (Table 6).

**Table 6:** Factors associated with passing neonatal resuscitation skills performance on first attempt

Variables	*N* (%) who passed OSCE		95% confidence interval		*p* value		95% confidence interval		*p* value
		CPR	Lower	Upper		APR	Lower	Upper	
Health facility level									
Health center	126 (94)	1.535	0.999	2.358	0.050	1.408	0.887	2.235	0.146
District hospital	173 (83.2)	0.846	0.606	1.180	0.323	0.774	0.536	1.118	0.172
Regional referral Hospital	121 (87.1)	Ref							
Regions									
Geita	211(90.6)	1.368	1.022	1.832	0.035	1.283	0.928	1.773	0.131
Shinyanga	209 (84.3)	Ref							
Work experience (years)									
≤ 5	130 (89)	1.344	0.917	1.970	0.129	1.004	0.526	1.917	0.990
6–10	196 (88.7)	1.319	0.932	1.867	0.118	1.081	0.643	1.815	0.769
> 10	94(82.9)	Ref							
Age (years)									
20–30	118 (87.4)	1.281	0.867	1.892	0.214	1.128	0.587	2.164	0.718
31–40	218 (89.7)	1.443	1.014	2.054	0.042	1.267	0.750	2.142	0.376
> 40	84 (81.6)	Ref							
Experience working in labor ward									
≤ 5	313 (87.4)	1.819	1.044	3.170	0.035	1.548	0.801	2.994	0.194
6–10	90 (90.9)	2.196	1.166	4.135	0.015	1.825	0.903	3.690	0.094
> 10	17 (70.8)	Ref							
Sex									
Male	167 (90.3)	1.271	0.938	1.724	0.122	1.032	0.732	1.457	0.856
Female	253 (85.5)	Ref							
Level of education									
Certificate	162 (82.7)	0.650	0.374	1.129	0.126	0.863	0.437	1.705	0.672
Diploma	215 (90.3)	0.932	0.534	1.627	0.804	1.143	0.599	2.182	0.685
Degree and above	43 (91.5)	Ref							
Position of the HCW									
Nurses	364 (86.5)	0.670	0.402	1.117	0.125	0.647	0.336	1.249	0.194
Doctors	56 (93.3)	Ref							

*CPR* crude prevalence ratio, *APR* adjusted prevalence ratio

## Discussion

In this study, we aimed to describe neonatal resuscitation skills acquisition after a 1-day simulation training using improved simulation tools, and associated factors, among healthcare providers working in the labour ward in facilities implementing SBBC intervention. We found that most participants (87.3%) passed the skills evaluation on their first attempt, with slight variation across facility levels, age, and experience working in the labor ward. However, detected variations were not statistically significant in regression modeling. Thus, participants in this study gained skills similarly regardless of their demographic characteristics.

Findings from this study suggest that a 1-day HBB curriculum-based training using improved training tools is still effective in imparting neonatal resuscitation skills to healthcare workers, as reported in previous studies [26, 27]. Skills acquisition in this study was comparable to other studies done in low-resource countries, which have shown adequate neonatal resuscitation skills acquisition after baseline simulation training. Most of these studies have shown that above 80% of the participants had adequate skills gains after initial training and had comparable OSCE scores [17, 28–32]. For instance, neonatal resuscitation skills evaluation at baseline before the countrywide implementation of HBB in Tanzania showed that 87.1% of healthcare providers passed skills evaluations, which is comparable to 87.3% in our study [33]. However, none of these studies used a live simulator and other Safer Births innovative tools in combination, as was done in this study. Thus, this study demonstrates that modern technological innovations for simulation training in neonatal resuscitation are feasible in low-resource settings where the use of innovative technological solutions for healthcare training is still limited. However, at this moment, we cannot tell if incorporating these innovative training tools will enhance clinical performance or skills retention among healthcare workers over time.

Notably, the effect of simulation is more marked when the training is done in a high simulation fidelity setting (physical and simulation tools that are close to real-life experience [34, 35]. Therefore, adequate skills gained in this study could have been attributed to using an improved neonatal simulator, which provides immediate feedback to the learner after the training session, coupled with visualization of “neonatal heart rate” during ventilation performance.

Other studies have demonstrated increased confidence and satisfaction of trainees while ensuring the safety of patients and learners [34, 36]. Simulation allows learners to improve their skills through repeated performance, with ample opportunity to correct errors and perfect skills without worry of endangering themselves and patients [36]. Additionally, simulation allows learners to improve in other interweaved cognitive and psychomotor skills, such as teamwork and communication skills, through role plays, feedback, and team debriefing [36, 37]. These attributes may have contributed to adequate skills acquisition in this study.

Our study did not find participants’ demographic characteristics independently associated with neonatal resuscitation skills acquisition. The region, facility level, age of participants, experience working in the labor ward, and level of education were found to influence passing OSCE evaluation; however, this influence was not significant in a multivariate model. This implies that healthcare workers acquired neonatal resuscitation skills using improved neonatal simulators and other tools similarly regardless of their differences in demographic characteristics and placement.

Similar studies that assessed providers’ characteristics associated with skills acquisition reported inconsistent findings; some did not show any association, while other studies reported significant associations with different providers’ characteristics, as shown in a systematic review of similar studies [38]. On the other hand, some studies have shown a similar trend to our study, where middle-level birth attendants perform better than lower or higher cadres [38]. Similarly, a study conducted in several regions in Tanzania and West Africa consistently showed that birth attendants working in higher facility levels, more urbanized areas, and high delivery volumes had relatively higher skill scores [39, 40].

The above-observed differences could be due to several reasons: HCWs working in higher volume facilities encounter more non-breathing neonates compared to those in low volume facilities since they receive referred pregnant mothers likely to experience complicated deliveries. Similarly, in Tanzania, middle-level attendants (registered nurses and midwives) conduct most routine deliveries and immediate neonatal resuscitation. In contrast, providers in low-level cadres perform direct supportive roles, whereas high-level cadres (physicians and graduate nurses) are rarely involved when consulted. Additionally, most highly learned providers are more involved in administrative duties, which do not provide them with enough opportunity to conduct deliveries and neonatal resuscitation. Besides, inconsistencies in similar studies could arise from using different statistical approaches to determine providers’ characteristics associated with skills performance.

Our study used the modified Poisson regression model to determine providers’ characteristics associated with skills performance. In contrast, most similar studies used the binary logistic regression model, which tends to overestimate the odd ratios when the likelihood of occurrence of the outcome of interest (passing) is greater than failing [24]. It is important to note that the HBB curriculum was designed to provide basic neonatal resuscitation skills that can be easily acquired by anyone attending deliveries at all levels, regardless of his/her training background and working experience [27]. Therefore, this study provides desirable results aligning with the objectives of the HBB curriculum whereby participants had comparable skills scores regardless of their background.

One major strength of our study is that we recruited many participants with different training backgrounds and experience, also working in other settings (rural, urban, peri-urban, and various levels of health service provision). This increases the generalizability of our study findings. Additionally, our statistical approach was more robust, which counteracts the overestimation of odd ratios; however, this study is limited by the fact that we did not assess the participant’s perspectives on the methodology and training tools, which might have influenced repeated skills practices after initial training. Moreover, we could not conduct a pre-training skills evaluation to compare with post-training scores as participants had no prior experience in using innovative tools used in this study. Therefore, we could not assess the difference in skills before the training. Furthermore, at this level, we cannot link the skills and clinical practice that eventually improve neonatal output.

## Conclusion

In conclusion, neonatal resuscitation skills training using technologically improved tools is feasible and effective in imparting skills among healthcare providers in resource-limited settings. Furthermore, healthcare providers gained skills similarly regardless of their educational background and work placement, which strengthens the suitability of the tools and the training modality.

Therefore, it is imperative that this intervention be scaled up in other facilities to improve healthcare providers’ skills in neonatal resuscitation and the provision of care for neonates. In addition, studies to assess skills retention after introducing low-dose, high-frequency simulation-based training using these improved tools are essential to ascertain if they are effective in skills retention with time and if this skill is translated to clinical practice and improved clinical outcomes of neonates.

## Data Availability

No datasets were generated or analysed during the current study.

## Abbreviations

CEmONC: Comprehensive Emergency Maternal, Obstetric and Newborn Care
HCW: Health care workers
HBB: Helping Babies Breathe
LMIC: Lower-and-middle-income countries
OSCE: Objectively Structured Clinical Evaluation
PAT: Pediatric Association of Tanzania
RHMT: Regional Health Management Teams
SBBC: Safer Births Bundle of Care
SDG: Sustainable Development Goals
SPSS: Statistical Package for Social Sciences
TAMA: Tanzania Midwives Association
